# Case Report: Effective Treatment With Pyrotinib and Capecitabine in a Heavily Pretreated Locally Advanced Breast Cancer Harboring Both HER2 Overexpression and Mutant

**DOI:** 10.3389/fonc.2021.715554

**Published:** 2021-10-14

**Authors:** Zhichao Gao, Junnan Xu, Yan Wang, Jie Wu, Tao Sun

**Affiliations:** ^1^ Department of Breast Medicine, Cancer Hospital of China Medical University, Liaoning Cancer Hospital and Institute, Shenyang, China; ^2^ Department of Breast Medicine, Key Laboratory of Liaoning Breast Cancer Research, Shenyang, China

**Keywords:** ERBB2 mutant, HER2 positive, L775S, pyrotinib, breast cancer

## Abstract

The prognosis for female patients with locally advanced breast cancer (LABC) has improved with the emergence of novel drugs, especially for those who have HER2 overexpression or ERBB-2 amplification. Trastuzumab-based regimen has been the paradigm in guidelines as first-line therapy, whereas many patients got progressive disease after several cycles of treatment or rapidly progress because of primary resistance. Point mutations of ERBB2 gene occur in both HER2-amplication and non-amplification patients, with a 2% ratio in HER2 non-amplification cohort and 1.48% in HER2 amplication population. The acquired mutation ratio of ERBB2 substantially raised to 16.7%–17.7% in patients prior to trastuzumab treatment. ERBB2 mutation may be a critical reason of resistance and disease progression among the patients treated with anti-HER2 monoclonal trastuzumab or dual anti-HER2 antibodies with trastuzumab and pertuzumab, or tyrosine-kinase inhibitor. ERBB-2 mutation with L755S and V842I indicates resistance to trastuzumab, while that with L755S and K753I indicates resistance to lapatinib; these mutations maybe sensitive to pan-HER tyrosine-kinase inhibitors. A 48-year woman diagnosed with HER2-positive LABC developed trastuzumab resistance after three lines of trastuzumab cross-line treatment with partial response (PR) as the best response. The tissue was performed by next-generation sequencing (NGS), and the results discovered L755S in ERBB2 gene. Then, she received effective treatment with pyrotinib plus capecitabine and underwent mastectomy after six cycles of combined treatment with PR. Subsequently, breast mastectomy was performed, and she took pyrotinib plus capecitabine for 1 year and pyrotinib monotherapy for another 1 year as adjuvant therapy and achieved a long-term clinical benefit. In conclusion, pyrotinib is a potential neoadjuvant agent for patients who are heavily pretreated and harbor both ERBB2 amplification and ERBB2 mutant in locally advanced breast cancer.

## Introduction

Breast cancer is the most common malignant tumor in women worldwide (1). Due to the prevalence of ultrasonography and mammographic screening, most patients are diagnosed at an early age; even the mass is untouchable, and the patients with early breast cancer had favorable prognosis (2). However, there are still a large number of patients who ignore the mass in their breast until it becomes huge, and the breast cancer cells invade the skin or chest, due to neglect or financial distress, especially in developing countries (2). Locally advanced disease indicates poor prognosis, poor opportunity of mastectomy, high risk of local relapse and distant metastasis (3). For locally advanced breast cancer (LABC), neo-adjuvant therapies are needed to reduce tumor size and lower stage and strive for the opportunity of surgery (3). The amplification of human epidermal growth factor receptor 2 (HER2, ERBB2) in breast cancer indicates high malignancy and rapid progression (4). Anti-HER2 target therapies are playing an important role in the treatment of HER2-positive LABC, and the prognosis has significantly improved with the emergence of novel anti-HER2 therapy. Currently, anti-HER2 drugs include the following three clinical categories: monoclonal bodies, such as trastuzumab, pertuzumab, and margetuximab; tyrosine-kinase inhibitors (TKI), such as lapatinib, neratinib, pyrotinib, and tucatinib; antibody drug conjugates (ADCs), such as trastuzumab emtansine (T-DM1) and trastuzumab deruxtecan (DS-8201).

Among the drugs mentioned above, trastuzumab-based regimen has been the paradigm in the first-line treatment of HER2-positive breast cancer since it was approved by Food and Drug Administration (FDA) in 1998. Most patients developed drug resistance during the first 12 months, and the resistance reasons were complex and had not been completely exposed (5, 6). ERBB2 mutation was suggested to be a potential reason that leads to resistance to trastuzumab-based regimen in several studies (7, 8). Approximately 3.9% of breast cancer patients harbor ERBB2 mutant, which causes constitutive activation of HER2 signal pathway and further leads to oncogenesis and tumor proliferation (8). Among various sites of mutation, an amino acid substitution from a leucine to a serine at position 755 in exon 19 in HER2 gene (L755S) is the most frequent hotpot, which has shown resistance to trastuzumab and lapatinib, but response to neratinib, an irreversible pan-HER TKI in previous studies (9, 10).

Similar to neratinib, pyrotinib is an irreversible pan-HER TKI that has achieved promising efficacy in number of clinical trials and real-world investigations (4, 11–14). In this study, we reported a case of LABC harboring both HER2 overexpression and mutant progression after three lines of trastuzumab-based regimen, in a patient who underwent radical mastectomy after preeminent PR response to pyrotinib and capecitabin as fourth-line treatment, and achieved a long-term clinical benefit from the constant treatment of pyrotinib plus capecitabin for 1 year and pyrotinib monotherapy for another 1 year as adjuvant therapy.

## Case Report

**Figure 1:**
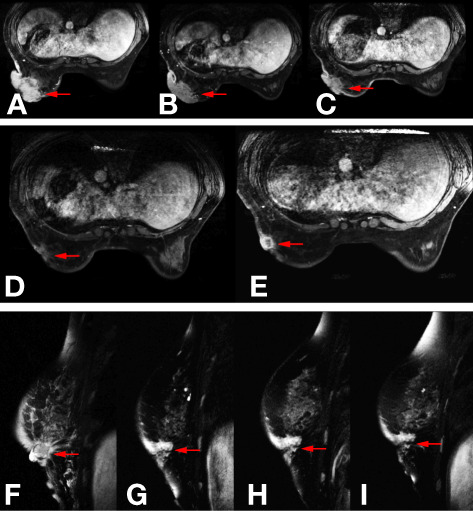
Images of mammary magnetic resonance imaging (MRI) in different phases. **(A)** The baseline mammary magnetic resonance imaging (MRI) in November 2017. **(B)** Mammary MRI after five cycles of TCbH regimen in February 2018. **(C)** Mammary MRI after four cycles of ECH regimen in May 2018. **(D)** Mammary MRI after two cycles of nab-paclitaxel plus trastuzumab regimen in July 2018. **(E, F)** Mammary MRI after four cycles of nab-paclitaxel plus trastuzumab regimen in August 2018. **(G)** Mammary MRI after two cycles of pyrotinib plus capecitabin regimen in October 2018. **(H)** Mammary MRI after four cycles of pyrotinib plus capecitabin regimen in November 2018. **(I)** Mammary MRI after six cycles of pyrotinib plus capecitabin regimen in December 2018.

**Figure 2:**
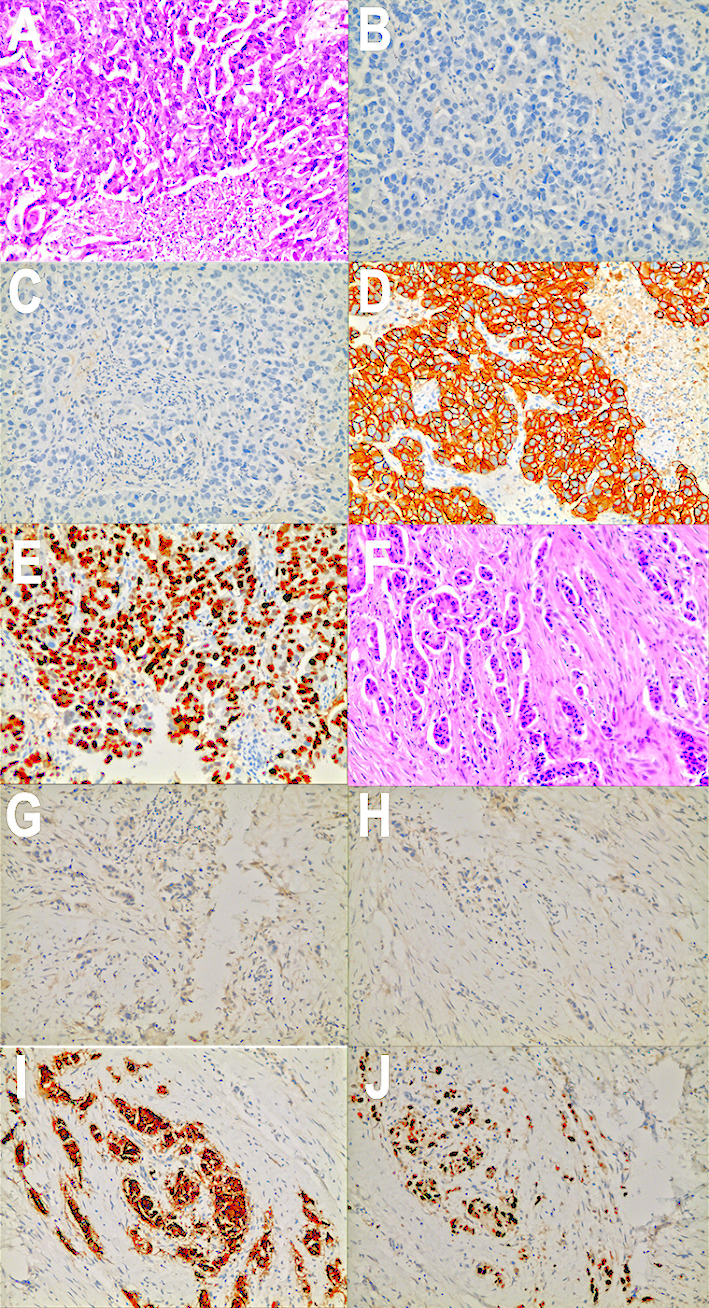
Pathology images and immunotherapy staining images of this patient. **(A)** Pathology images of core needle biopsy in October 2017. **(B)** ER immunotherapy staining image of core needle biopsy in October 2017. **(C)** PR immunotherapy staining image of core needle biopsy in October 2017. **(D)** HER2 immunotherapy staining image of core needle biopsy in October 2017. **(E)** Ki-67 immunotherapy staining image of core needle biopsy in October 2017. **(F)** Pathology images of mastectomy in January 2019. **(G)** ER immunotherapy staining image of mastectomy in January 2019. **(H)** PR immunotherapy staining image of mastectomy in January 2019. **(I)** HER2 immunotherapy staining image of mastectomy in January 2019. **(J)** Ki-67 immunotherapy staining image of mastectomy in January 2019.

The 48-year-old woman unintentionally felt a painless mass of about 2 cm × 1 cm in the upper outer quadrant of her left breast in March 2017, which gradually enlarged due to her neglect. In September 2017, the mass rapidly enlarged to 7cm × 6 cm, and there was infiltrated erythema and ulceration on the skin of the breast. In October 2017, she was admitted to our hospital and received mammary magnetic resonance imaging (MRI) (**Figure 1A**), which showed a 91 mm × 55 mm × 87 mm mass with ill-defined margins and a heterogeneous internal enhancement in the left breast; breast imaging reporting and data system (BI-RADS) category was identified as 5. Additionally, enlarged lymph nodes in her left axilla were observed. Laboratory examinations presented normal carcinoembryonic antigen (CEA) level (0.926 ng/ml) and high cancer antigen 15-3 (CA15-3) level (61.4 U/ml). Subsequently, core needle biopsy was performed on October 28, 2017, and invasive ductal carcinoma was pathologically diagnosed, histological grade 2, with estrogen receptor (ER) negative and progesterone receptor (PR) negative, overexpression of HER-2 (+++), and high proliferation index Ki-67 (70%) (**Figures 2A–E**). The patient was in good health without any medical history of hypertension or diabetes and without smoking, drinking, and other bad habits. She had menarche at 13 years old, and the last menstrual period was October 11, 2017. Before any treatment, we offered a thorough examination and excluded any distant metastases. The clinical stage was cT4N2aM0 according to the 7th edition of American Joint Committee on Cancer (AJCC) staging manual (15).

As pertuzumab and T-DM1 had not been approved in China at that time, after obtaining informed consent, we adopted a TCbH regimen (docetaxel, 75 mg/m^2^ d1; carboplatin, AUC = 6 d1; trastuzumab, 8 mg/kg for the first cycle and 6 mg/kg thereafter d1, 21 days/cycle) as first-line treatment according to the National Comprehensive Cancer Network (NCCN) guidelines. After five cycles (November 6, 2017–January 30, 2018), reexamination of mammary MRI (**Figure 1B**) showed the size of the mass shrank from 91 mm × 55 mm × 87 mm to 84 mm × 48 mm × 72 mm. According to the evaluation criteria of Response Evaluation Criteria in Solid Tumors (RECIST) version 1.1, we judged the therapeutic effect as stable disease (SD).

**Figure 3:**
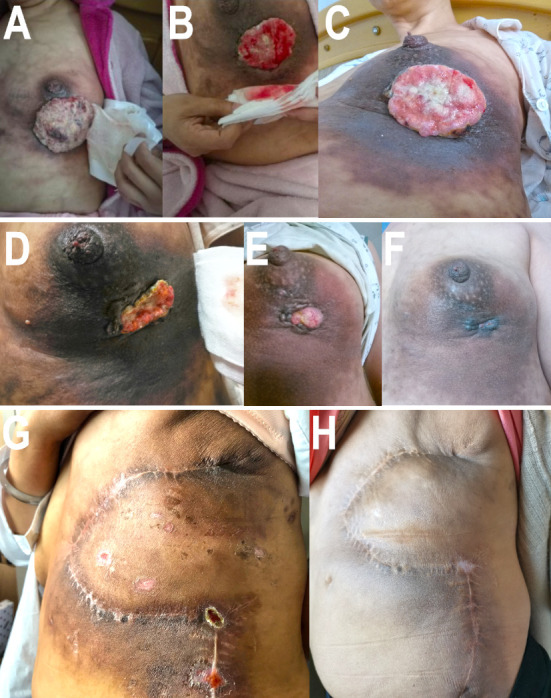
Appearances of breast or chest wall in different phases. **(A)** The appearance of breast before ECH regimen in March 2018. **(B)** The appearance of breast after two cycles of ECH regimen in April 2018. **(C)** The appearance of breast after four cycles of ECH regimen in May 2018. **(D)** The appearance of breast before pyrotinib plus capecitabin regimen in August 2018. **(E)** The appearance of breast after two cycles of pyrotinib plus capecitabin regimen in October 2018. **(F)** The appearance of breast after six cycles of pyrotinib plus capecitabin regimen in October 2018. **(G)** The appearance of left chest wall 5 months after surgery in June 2019. **(H)** The appearance of left chest wall 26 months after surgery in March 2021.

Based on the NOAH test (16), limited cycles of joint application of trastuzumab and anthracycline were effective and had controllable cardiac toxicity. Therefore, we choose ECH (epirubicin, 90 mg/m^2^ d1; cycloposphamide, 600 mg/m^2^ d1; trastuzumab, 6 mg/kg d1, 21 days/cycle) regimen as second-line treatment (February 27, 2018–May 23, 2018). After four cycles, re-examined mammary MRI (**Figure 1C**) showed that the tumor shrank from 84 mm × 48 mm × 72 mm to 66 mm × 29 mm × 56 mm. According to the evaluation criteria of RECIST v1.1, the therapeutic effect was still SD. There was no significant change in the appearance of the breast (**Figures 3A–C**).

As the efficacy still did not reach PR, the regimen was altered to nab-paclitaxel combined with trastuzmab. For metastatic breast cancer patients who had received prior taxanes, nab-paclitaxel plus still has the objective response rate of 54.2% (17), and nab-paclitaxel plus traszumab to EC was superior to taxanes combined with trastuzumab to EC as neo-adjuvant therapy in GBG 69 trial (18). Therefore, we choose nab-paclitaxel plus trastuzumab as the third-line treatment (nab-paclitaxel, 125 mg/m^2^ d1, d8; trastuzumab, 6 mg/kg d1, 21 days/cycle). The patient got PR after the first two cycles (**Figure 1D**), but two cycles later, the efficacy deteriorated to progressive disease (PD) (**Figure 1E**).

**Figure 4:**
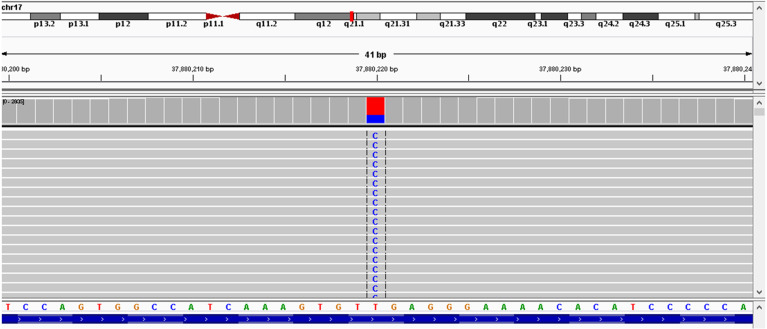
Next-generation sequencing (NGS) showed L755S mutation in exon19 of ERBB2 gene.

As the patient had developed acquired trastuzumab resistance after three lines of trastuzumab-based regimen, another regimen with distinct mechanism of anti-HER2 therapy was needed. To investigate the potential reason of drug resistance, a next-generation gene sequencing (NGS) of 425-gene panel was performed using the tissue repunctured from the tumor. According to the NGS report, persistent existence of ERBB2, CDK12, and MYC amplification with abundance of 15.7, 16.2, and 7.6 were detected, respectively. Meanwhile, there were mutations as p.R314H on IDH1 gene and p.H179R on TP53 gene. On ERBB2 gene, the patient had L755S (**Figure 4**) and K907R mutation. L755S mutation indicated resistance to trastuzumab and lapatinib (9, 10) and may be sensitive to neratinib (10), an irreversible pan-HER TKI. At that time, neratinib had not been admitted to China, and a similar agent, pyrotinib, had just been approved by the China Food and Drug Administration (cFDA), whose phase II trial had indicated that for the treatment of advanced HER2-positive breast cancer patients, pyrotinib combined with capecitabine had better efficacy than lapatinib combined with capecitabine (12). Thus, we selected pyrotinib plus capecitabine (pyrotinib, 400 mg/d d1–21; capecitabine, 1,250 mg/m^2^ bid d1–14, 21 days/cycle) as the fourth-line treatment regimen. After six cycles (August 28, 2018–December 29, 2018), the patient got PR (**Figures 1F–I**), and the surface of the breast almost completely healed (**Figures 3D–F**).

Left breast cancer mastectomy and local rotation skin flap grafting were performed on the patient in January 16, 2019, and postoperative pathology report showed an invasive ductal carcinoma with changes in Miller–Payne Grade 4 (**Figure 2F**), with a tumor size of 1.0 cm × 0.8 cm × 0.8 cm and lymph nodes negative (0/14). Immunohistochemistry indicated ER (−), PR (−), C-erbb-2 (+++), and ki-67 (70%+) (**Figures 2G–J**).

The patient continued to receive pyrotinib plus capecitabine (pyrotinib, 400 mg/day d1–21; capecitabine, 1,250 mg/m^2^ bid d1–14, 21 days/cycles) synchronous with radiotherapy (left chest wall plus left supraclavicular area, 5,000 cGy/25f), and pyrotinib plus capecitabine continued to be administrated for 1 year. There were no signs of metastasis or recurrence after an overall examination after 1 year of adjuvant therapy with pyrotinib plus capecitabine. The patient suspended capecitabin due to severe hand–foot syndrome and took pyrotinib as single agent for another 1 year (**Table 1**), partially referred to ExteNET clinical trial (19). By the time of contribution, the second (June 2019) and last regular follow-ups (March 2021) showed no signs of metastasis or recurrence (**Figures 3G, H**).

**Table 1:** The course of disease in the patient diagnosed with breast cancer.

Time frame	Line of treatment	Therapy regimen	Response
Nov 2017–Jan 2018	First-line	Docetaxel, carboplatin, trastuzumab × 5 cycles	Stable disease (SD)
Feb 2018–May 2018	Second-line	Epirubicin, cyclophosphamide, trastuzumab × 4 cycles	Stable disease (SD)
May 2018–Aug 2018	Third-line	Nab-paclitaxel, trastuzumab × 4 cycles	Partial response (2 cycles); progression disease (4 cycles)
Aug 2018–Dec 2018	Fourth line	Pyrotinib, capecitabine × 6 cycles	Partial response (PR)
Jan 2019	Mastectomy	Radical resection plus local rotation skin flap grafting	Complete response (CR)
Feb 2019–Feb 2020	Adjuvant	Pyrotinib, capecitabine	no recurrence/metastasis
Feb 2020–Feb 2021	Adjuvant	Pyrotinib	no recurrence/metastasis

## Discussion

About 20% of breast cancers have HER2 overexpression or ERBB2 amplification, and patients who are HER2 positive have poor prognosis, especially without anti-HER2 target therapy (20). As a transmembrane tyrosine kinase receptor, HER2 overexpression can combine with extracellular ligand and activate downstream signaling pathways such as PI3K/AKT and Ras/Raf/MEK and promote tumor proliferation. Trastuzumab-based therapy has become the standard regimen recommended by the NCCN guidelines for the treatment of locally advanced or metastatic breast cancer (21). However, trastuzumab often shows primary or acquired resistance during or posttreatment, and their resistance mechanisms include p95 overexpression in HER2 (truncated HER2) (22), abnormal activation of downstream PI3K/AKT/mTOR pathways (23), overexpression of insulin-like growth factor 1 receptor (IGF-1R) (24), and so on. In recent years, ERBB2 mutant has been a new hotpot as a potential reason of resistance to trastuzumab-based regimen in several studies (7, 8).

As is mentioned above, ERBB2 mutation can be identified in nearly 4% of breast cancer patients, with a 2% ratio in HER2-negative cohort and 1.48% in HER2-amplification cohort (25, 26). But among prior trastuzumab-treated patients, the ratio of ERBB2 mutation substantially raised to 16.7%–17.7%, which may be an acquired resistance mutation (27, 28). Among all the single-nucleotide polymorphisms (SNPs) discovered, L755S was the most frequent spot (9). Certain somatic ERBB2 mutation leads to constant activation of tyrosine kinase, which can subsequently activate downstream signal pathway independent of receptor dimerization extracellular and induce breast cancer cell proliferation and metastasis (29). ERBB2 mutations were heterogeneous and presented varying effects on anti-HER2 therapy. Mutation with L755S and V842I indicated resistance to trastuzumab, while L755S and K753I indicated resistance to lapatinib (30). For HER2-positive patients who harbored L755S mutation, the SUMMIT trail indicated that neratinib was the most effective agent (8) and improved survival in this population. For the patient in this passage who carried L755S mutation in ERBB2 gene, as neratinib had not be approved in China at that time, pyrotinib, which has similar molecular mechanism with neratinib, was the optimal choice and had shown great effects. K907R mutation, which coexisted with L755S for the patient, had not been proven as a predictive marker in previous studies. In addition, trastuzumab resistance can also be caused by certain HER-2 variants, such as the deletion of exon 16 (d16HER-2) and a subtype that expresses a series of carboxyterminal named as p95HER-2. In previous studies, D16HER-2 and p95HER-2 indicated more aggressiveness, with worse prognosis than those with wild-type isoform (31, 32). D16HER-2 was sensitive to HER-2 TKIs, as lapatinib and neratinib, while p95HER-2 was sensitive to anti p95HER-2 antibodies. Some studies also indicated that truncated p95-HER2-expressing cells retain their kinase activity and are responsive to lapatinib therapy (33).

For advanced HER2-positive breast cancer, patients can benefit from continuous anti-HER2 therapy (34). However, for the treatment after resistance of trastuzumab, cross-line treatment of trastuzumab based regimen can still benefit some patients, especially at that time with limited anti-HER2 agents. GBG 26 study included 156 patients who had progressed after trastuzumab-based regimen as first-line treatment and were included in the treatment group receiving trastuzumab combined with capecitabine or capecitabine monotherapy at 1:1 ratio (34). The results showed that the combined group had significant advantages in PFS (8.2 vs. 5.6 months, p = 0.0338) and OS (25.5 vs. 20.4 months, p = 0.257). In HERMINE study (35), 623 HER2-positive breast cancer patients, who had progressed from trastuzumab-contained regimens as first-line treatment, were randomly assigned to the following two groups: one cohort with continuing use of trastuzumab for 30 days or more, and another group who stopped using trastuzumab before progression or received trastuzumab for 30 days or less after progression. According to the results at 2 years follow-up, time to progression (TTP) (10.2 vs. 7.1 months, p = 0.0215) and OS (27.8 vs. 16.8 months, p < 0.001) of the long-term use of trastuzumab after progression of first-line treatment group were significantly superior to that of the stopped group. All the studies above have indicated that trastuzumab is a reasonable and effective choice for cross-line therapy, especially before the listing of new drugs, such as neratinib and pyrotinib. Based on the results, we adopted trastuzumab cross-line treatments as the second- and third-line regimens.

Lapatinib is a small-molecule epidermal growth factor receptor (ErbB1) and human epidermal factor receptor 2 (ErbB2) tyrosine kinase inhibitor. The listing research EGF100151 showed that lapatinib combined with capecitabin extended 4.0 months of TTP and had a 51% lower risk of disease progression than capecitabin alone after resistance to trastuzumab (36). EGF104900 research indicated that lapatinib plus trastuzumab was also a considerable choice for patients who has intolerance to chemotherapy and resistance to trastuzumab (37). In this case, we did not choose lapatinib for financial distress and the distinct mechanism with pyrotinib of the patient.

In recent years, with new targeted drugs emerging constantly, the prognosis has been improved and the layout of anti-HER2 have gradually changed. More and more novel options can be chosen for treatment after trastuzumab resistance, and the efficacies of lapatinib and trastuzumab cross-line regimens seem to be humble. Pertuzumab can inhibit dimerization of HER2, which is complementary to trastuzumab. The study of BO17929 showed that patients with trastuzumab resistance could also obtain 24.2% overall response rate (ORR) and 50% CBR by using the double-targeted therapy of pertuzumab combined with trastuzumab (38). Margetuximab is a chimeric, Fc-engineered, immune-activating anti-ERBB2 immunoglobulin G1 (IgG1) monoclonal antibody. The phase III SOPHIA trial showed that for HER2-positive advanced breast cancer patients who had progressed from two or more prior anti-ERBB2 therapies, margetuximab plus chemotherapy resulted in statistically significant improvement in PFS (5.8 vs. 4.9 months) than trastuzumab plus chemotherapy (39). In addition, T-DM1, an antibody–chemotherapy drug coupling agent, is playing an increasingly crucial role in the treatment of advanced HER2-positive breast cancer. TH3RESA research indicated that in third-line treatment of HER2-positive advanced breast cancer, T-DM1 can significantly improve PFS (6.2 vs. 3.3 months, p < 0.001) and OS (22.7 vs. 15.8 months, p= 0.0007) (40). EMILIA research showed that T-DM1 had significant advantages in PFS (9.6 vs. 6.4 months, p < 0.001), OS (30.9 vs. 25.1 months, p < 0.001), and ORR (43.6% vs. 30.8%) than lapatinib combined with capecitabine for the patients who had trastuzumab resistance (41). As T-DM1, Trastuzumab deruxtecan (DS-8201) is also an antibody–drug conjugate, which is composed of an anti-HER2 antibody and a cytotoxic topoisomerase I inhibitor. In Destiny-Breast01 research, DS-8201 showed a revolutionary breakthrough of efficacy with an ORR of 60.9% and a median progression free survival (mPFS) of 16.4 months in heavily pretreated HER2-positive patients (42). For this patient, dual-targeted therapy of pertuzumab plus trastuzumab plus chemotherapy, margetuximab plus chemotherapy, T-DM1, and DS-8201a are all rational choices if recurrence or metastasis occurs in the future. Neratinib is a pan-HER irreversible tyrosine kinase inhibitor. A phase II clinical trial showed that second-line neratinib monotherapy was not inferior to lapatinib combined with capecitabin in patients who had previously received trastuzumab (43). However, the unavailability of DS8201a and margetuximab and the high price of T-DM1 and neratinib in China greatly limited the clinical application of these drugs.

As neratinib, pyrotinib is an irreversible TKI that has anticancer effects by blocking EGFR, HER2, and HER4 signaling pathways. Pyrotinib can directly act on the tyrosine kinase region of the HER2 pathway and comprehensively block all downstream dimers including HER2 homodimers and heterodimers. Compared with lapatinib, the targets of pyroitinib are more comprehensive, and the inhibition is irreversible. Compared with neratinib, pyroitinib has higher bioavailability, which stands for higher inhibition intensity to tumor cells and has tolerable safety (44). Phase II clinical trials showed that, for patients with HER2-positive advanced breast cancer, regardless of previous trastuzumab treatment, pyroitinib combined with capecitabine was superior to lapatinib combined with capecitabine in terms of PFS (18.1 vs. 7.8 months) and ORR (78.5% vs. 57.1%) (12). In the subgroup of trastuzumab pretreatment, the median PFS in the pyrotinib-combined group had not been achieved, but there had been a significant difference between the two groups. PHENIX clinical trial published by the American Society of Clinical Oncology (ASCO) in 2019 further confirmed that pyrotinib combined with capecitabin was a safe and effective choice for trastuzumab resistance, with mPFS of 11.1 months and mORR of 68.6% (12). In each subgroup, pyrotinib-combined regimen showed better efficacy. Moreover, after unblinding, 5.5 months of mPFS and 38% ORR were obtained in the control capecitabine monotherapy group to receive pyrotinib monotherapy after progression. In phase III PHOEBE study, pyrotinib plus capecitabine showed greater benefits than lapatinib plus capecitabine in mPFS (12.5 vs 6.8 months, p < 0.0001) (11).

For this patient, after the failure of trastuzumab cross-line treatments, the combined treatment of pyrotinib and capecitabine showed obvious tumor regression, strived the opportunity of surgery, and offered a long-term clinical benefit for the patient. To the authors’ knowledge, there is no standard regimen for adjuvant therapy of patients in this category, including NCCN guidelines, ABC5 guidelines and Chinese Society of Clinical Oncology (CSCO) guidelines. Considering that the patient has benefitted significantly from pyrotinib combined with capecitabine, the original treatment regimen was continued, and regular follow-ups were conducted.

## Conclusion

For HER2-positive LABC, trastuzumab-based regimen is the standard treatment, but a number of patients developed drug-resistance for ERBB2 mutant. In certain cases, as L755S mutation, pyrotinib is a reliable agent that provides a safe and effective treatment option. Its remarkable curative effect changed the strategy of treatment in patients with advanced HER2-positive breast cancer. By the time, pyrotinib has been approved as the second-line treatment of HER2-positive advanced breast cancer by CFDA. We expect that more specific and suitable patients benefit from pyrotinib in the neo-adjuvant/adjuvant settings.

## Data Availability Statement

The original contributions presented in the study are included in the article/supplementary material. Further inquiries can be directed to the corresponding author.

## Ethics Statement

The studies involving human participants were reviewed and approved by Human Ethics Review Committee of Liaoning Cancer Hospital and Institute, with the Ethical Approval Number: 20200752. The patients/participants provided their written informed consent to participate in this study. Written informed consent was obtained from the individual(s) for the publication of any potentially identifiable images or data included in this article.

## Author Contributions

ZG and JX performed the literature search and drafted the manuscript. YW and JW offered photos and imaging data. TS designed and supervised the study. All authors contributed to the article and approved the submitted version.

## Funding

This work was supported by CSCO Project (Y-HR2018-362, TS), Wujieping Project (320675018541, TS), Liaoning Cancer Hospital Yangtse River Scholars Project (JX), Medical-Engineering Cross Research Fund between Liaoning Cancer Hospital and Dalian University of Technology (LD202022, TS), and Liaoning Revitalization Talents Program (XLYC1907160, JX).

## Conflict of Interest

The authors declare that the research was conducted in the absence of any commercial or financial relationships that could be construed as a potential conflict of interest.

## Publisher’s Note

All claims expressed in this article are solely those of the authors and do not necessarily represent those of their affiliated organizations, or those of the publisher, the editors and the reviewers. Any product that may be evaluated in this article, or claim that may be made by its manufacturer, is not guaranteed or endorsed by the publisher.
